# A ‘Real Life’ Service Evaluation Model for Multidisciplinary Thyroid Eye Services

**DOI:** 10.3389/fendo.2021.669871

**Published:** 2021-05-07

**Authors:** Soma Farag, Claire Feeney, Vickie Lee, Sonali Nagendran, Rajni Jain, Ahmad Aziz, Rashmi Akishar, Vassiliki Bravis, Karim Meeran

**Affiliations:** ^1^ Imperial College School of Medicine, Imperial College London, London, United Kingdom; ^2^ Department of Metabolism, Digestion and Reproduction, Imperial College London, London, United Kingdom; ^3^ Department of Ophthalmology, The Western Eye Hospital, Imperial College Healthcare National Health Service (NHS) Trust, London, United Kingdom; ^4^ Department of Ophthalmology, Central Middlesex Hospital, London North West Healthcare NHS Trust, London, United Kingdom

**Keywords:** thyroid-associated ophthalmopathy, Graves ophthalmopathy, optic neuropathy, Graves disease, Graves orbitopathy, thyroid eye disease

## Abstract

**Background/Aims:**

There is no universal consensus on the practical implementation and evaluation of the Amsterdam Declaration on Graves Orbitopathy in a Multidisciplinary Thyroid Eye Disease (MDTED) pathway. Recent recommendations from the UK TEAMeD-5 and BOPSS initiative highlight the importance of prevention, screening, and prompt referral of patients with moderate to severe and sight-threatening thyroid eye disease to multidisciplinary (MDTED) clinics and recommends annual auditing. We propose a practical service evaluation model with Key Performance Indicators (KPI) that are achievable and could be implemented across most TED pathways.

**Material and Methods:**

We conducted a service evaluation from an integrated TED pathway in London with three MDTED clinics. Data was collected retrospectively from consecutive TED patients included: 1) Patient demographics, 2) Referral to first appointment time, 3) Documented smoking cessation and selenium supplementation advice, 4) Presenting disease activity and severity, 5) Investigations and treatments, including radio-iodine, 6) Time from decision to treatment initiation, 7) Initial and subsequent thyroid status.

**Results:**

The median age was 49.0 yrs, 77.5% (183/236) were female and 49.5% (101/204) Afro-Caribbean or Asian. At their first clinic attendance, 47.6% (110/231) were biochemically euthyroid and 76.7% (79/103) at discharge. All 23.1% (52/225) current smokers received smoking cessation advice and 64.8% (153/236) received selenium supplementation advice. Intravenous methylprednisolone was given to 33.9% (80/236) patients and 12.7% (30/236) received second-line immunosuppression. All 7.2% (17/236) patients with sight-threatening disease received treatment within two weeks of diagnosis.

**Conclusions:**

This study forms a waymark for other units using TEAMeD-5 and BOPSS audit criteria. Dedicated electronic patient records with ongoing data capture, including quality of life assessments, and diagnostic coding would significantly aid future auditing, improve patient care, and facilitate a national audit of TED management. A future survey when the TED standards have become embedded would be instructive to see whether this has improved TED care.

## Introduction

Thyroid Eye Disease (TED) is a distressing and sometimes sight-threatening complication of Graves’ Disease (GD) which affects up to 400,000 people in the UK and an estimated 9 per 10,000 population in Europe (1, 2). Women are more commonly affected than men (1, 3). There have been several European initiatives including Amsterdam Declaration of 2009 (3) and the 2016 European Group on Graves Orbitopathy (EUGOGO) recommendations for timely therapeutic interventions in order to optimise outcomes (4, 5).

In response TEAMeD-5 (Thyroid Eye Disease Amsterdam Declaration Implementation Group) was launched in 2017 (6) to raise awareness among endocrinologists and patients with GD about the importance of prevention, early detection and prompt referral of moderate to severe and sight-threatening TED to a specialist multidisciplinary thyroid eye disease (MDTED) clinic (4). TEAMeD-5 recommended mainly endocrinological Key Performance Indications (KPIs). In 2019, TEAMeD and the British Oculoplastic Surgery Society (BOPSS) published further recommendations based on a national survey of TED services and that were endorsed by all the UK TED stakeholder organizations. In our study, we aimed to carry out a service evaluation of a large multi-disciplinary TED network in a multi-ethnic metropolitan setting, as a waymark for implementation of the aforementioned recommendations.

## Material and Methods

### Patient Recruitment

We conducted a retrospective observational study of patient databases from three MDTED clinics led by consultant ophthalmic and oculoplastic surgeons (VL, RJ and AA), and endocrinology consultants (including KM VB and CF). The patients’ care also included orthoptists who assessed for ocular motility disturbance and an immunosuppression specialist (RA), for patients requiring second-line immunosuppression. There is an integrated network of other specialists including head and neck radiologists, radiotherapists for orbital radiotherapy, thyroid surgeons for thyroidectomy and an elective rehabilitation pathway (including orbital decompression ocular muscle surgery for diplopia and eyelid surgery) for patients with quiescent residual disfiguring and debilitating disease.

We formulated the KPIs used in this study from the 2019 recommendations of the British Oculoplastic Surgery Society (BOPSS) and TEAMeD-5 (**Table 1**) (7).

**Table 1:** The British Oculoplastic Surgery Society proposed audit criteria for the review of services managing thyroid eye disease (6) and the findings of this study.

i)	BOPSS audit criteria	Audit results
***(1) Efficacy***	Consideration of oral selenium supplements for patients with mild, active TED.	Out of the 135 new patients seen after the introduction of TEAMeD-5 in 2017, 60.0% (81/135) were advised to take oral selenium supplements.
	Smoking cessation advice for patients who are smokers.	The 23.1% (52/223) documented smokers in our cohort 100% received smoking cessation advise.
	Prompt correction of dysthyroidism and maintenance of euthyroidism.	Not formally audited.
		76.7% (79/103) were biochemically euthyroid at discharge from MDTED clinic
	Where systemic steroid treatment is indicated, use of intravenous pulses of methylprednisolone in preference to oral steroids.	Departmental SOP is IVMP only for first line immunosuppression. No patients had oral prednisolone as first-line treatment.
		80/236 (33.9%) of our patients received IVMP.
	Number receiving urgent treatment for sight-threatening orbitopathy including surgical decompression for patients who fail to respond to high dose intravenous steroids.	7.20% (17/236) received the EUGOGO protocol of 3-day high dose IVMP for sight-threatening orbitopathy followed by the standard 12 week course.
		9 of these 17 patients also underwent emergency decompression surgery.
	Prevalence of patients treated orbital irradiation.	38/236 (16.1%) patients received orbital irradiation.
	Patients undergoing elective orbital decompression and rehabilitative surgery for patients with inactive or minimally active disease who are significantly impaired (socially or psychologically) as a result of TED.	16/236 (6.78%) patients underwent elective orbital decompression as part of rehabilitative surgery for TED.
***(2) Safety***	Patient education for recognising side-effects of steroids and other immunosuppressive treatments.	Our patients receive information leaflets provided by the local trust and leading TED patient charities on the side effects of steroid and immunosuppressive medication.
		12.7% (30/236) of our patients received second-line immunosuppression under care of the MDTED immunosuppression specialist.
	Appropriate selection of patients with TED who are being considered for radioiodine for suitability of steroid cover.	There is a departmental SOP for RAI treatment, with recommendations for adjunctive oral steroid cover for at risk patients.
		25% (2/8) patients under our MDTED clinic received steroid cover. The remainder were sent for RAI suitability screening.
	Safe use of immunosuppressive treatments (exclusion of those for whom there are contraindications, assessment, and monitoring of risks of serious adverse effects	100% (80/80) of those requiring intravenous steroids were given in the day unit of a large teaching hospital with acute services.
		100% (30/30) of those requiring second-line immunosuppression received this under the care of an immunosuppression specialist who is part of the MDTED clinic.
	Timely assessment of response to high dose intravenous steroids and withdrawal of steroid treatment in favour of other therapies for those with inadequate response.	There is a SOP that patients are seen monthly in MDT clinic and their treatment response is assessed, with a personalised management plan formulated. Our departments compliance with SOP was not formally audited
***(3) Patient-centred care***	Availability of good quality information about GO, its usual course, likely outcomes, and potential treatments, complemented by high quality written information and access to patient-led organisations.	100% (236/236) given written information on TED.
	Formulation of personalised management plans following multidisciplinary discussion.	100% (236/236) of the patient management plans are discussed with consultant Endocrinologist and Ophthalmologists at every visit. A board round is conducted at the end of each clinic to ensure a consensual and personalised management plan for each patient.
	Good communication between the clinical team and the patient.	100% (236/236) patients have their management plans discussed with them and are sent copies of clinic letters.
	Patient engagement with the decision process about management of TED.	
	Use of validated tools to assess the impact of GO on their quality of life.	Our study found that there was no uniform QOL collection as a result of this finding the departmental SOP was changed to ensure MDTED patients have a GOQOL assessment at each clinic visit
***(4) Timely***	Patients with sight-threatening TED (dysthyroid optic neuropathy resulting in significant reduction in visual acuity, corneal breakdown with impending or established infection, globe subluxation) should be treated urgently within 2 weeks	100% (17/17) achieved this.
		Median (range) decision to treatment time was 1.6 (0-6) days.
	Patients with moderate-to-severe, active TED should be offered treatment within six weeks from presentation	96.3% (77/80) received treatment within six weeks from presentation.
	Multiple surgical treatments in patients requiring complex rehabilitative surgery, should follow the sequence: orbital decompression/eye muscle surgery/lid surgery	This sequence was not audited, however it is in the departmental SOP.
***(5) Efficient***	Referral pathways from primary to secondary and tertiary care, should be well-defined and seamless	75.2% (177/236) were seen in a MDTED clinic within three months of referral.

BOPSS, British Oculoplastic Surgery Society; IVMP, Intravenous methylprednisolone; MDTED, Multidisciplinary Thyroid Eye Disease Clinic; RAI, Radio-iodine; SOP, Standard operating protocol; TED, Thyroid Eye Disease.

Patients were identified from MDTED clinic databases at Imperial College Healthcare NHS Trust and Central Middlesex Hospital (CMH) (part of London North West University Healthcare NHS Trust). Data was collected at a contemporaneous post clinic ‘board round’ consensus of all the MDT consultants, who are also authors of this paper.

Additional clinical data was collected from the electronic record systems and included clinic letters, biochemical and radiology results. All patient data was collected from inception of the MDT clinic. The duration of the service evaluation started from the inception of the MDT clinics - CMH (Nov 2011), Imperial College sites at the Western Eye Hospital (WEH) Jan 2016) and Charing Cross Hospital (CXH) Jan 2017).

Database lock was on 31st January 2019, which was six months before the initiation of analysis.

This study was registered and approved by the Audit Department of the Trusts. This study adhered to the tenets of the Declaration of Helsinki.

### Clinical Assessment

All TED patients had a Clinical Activity Score (CAS) assigned by a consultant ophthalmologist at each visit. A CAS of ≥ 3 defined clinically active TED, although other factors such as proptosis, ocular motility disturbance and optic nerve compromise were also taken into account. The severity of the disease was graded according to the EUGOGO categories of disease severity (mild, moderate to severe and sight-threatening) (4). All patients with a CAS of 3 or above were classified as having at least moderate disease severity. MRI was the imaging modality of choice as it offers superior soft tissue resolution making it an appropriate assessment of TED, which is a soft tissue disease. First-line immunosuppression was once weekly intravenous methylprednisolone (IVMP) infusions given over 12 weeks, as recommended by the EUGOGO consensus (4). All patients had at least one endocrine assessment, with optimisation of thyroid status, where appropriate. In the majority of patients with hyperthyroidism, the treatment was either antithyroid medication (Carbimazole first-line and Propylthiouracil second-line), thyroxine replacement, or both (i.e., ‘block and replace’). Hypothyroid patients and post thyroidectomy patients were treated with thyroxine replacement guided by their thyroid function.

The following data was retrospectively collected for each patient:

1. Demographic data: age, gender, ethnicity, family history (in first or second-degree relatives) of thyroid disease, smoking status (current, ex-smoker, never smoked), diabetes mellitus (type 1 and 2)
2. Endocrine data:
  - Thyroid status at first MDTED clinic visit
  - Management of thyroid disease - treatment (drugs, radioiodine (RAI), thyroidectomy), endocrine control and relapse of thyroid dysfunction
  - Initial thyroid status of patients at referral and during period of treatment and at the last follow-up visit
  - Number of patients who received RAI treatment
  - TSH-R antibody status
3. MDTED clinical metrics data:
  - Time between referral and a specialist review at first MDTED clinic
  - Disease activity (determined by CAS at the first and last MDTED clinic). Patients with a CAS ≥3 were deemed to have active disease.
  - Disease severity (determined by a clinical assessment) in line with EUGOGO classification
  - Outcome of patients referred to MDTED clinic (with a confirmed diagnosis of TED), including investigations and treatment given
4. Management of TED:
  - Duration of treatment for TED: this was estimated from the patient reported date of TED onset, date of referral and of first MDTED clinic
  - Number of patients receiving documented advice on smoking cessation and selenium supplementation
  - Time from decision to treat to initiation of treatment for moderate to severe and sight-threatening active disease. Time from decision to treatment was recorded for: IVMP infusions, orbital decompression (urgent and elective) and orbital radiotherapy.

### Inclusion and Exclusion Criteria

Inclusion criteria:

- At least 18 years of age
- A diagnosis of TED (any severity, active or inactive)

Exclusion criteria:

- Seen at MDTED, where a diagnosis of TED was excluded
- Where the patient’s endocrinology care was not at Imperial or CMH and where it was not possible to access their blood results or advise on their endocrine care.

### Statistical Analyses

Statistical analyses were performed using IBM SPSS statistics v.25 and GraphPad Prism v.8. The demographic data are presented as number and percentage. Descriptive statistics for non-normally distributed data used median and inter-quartile range.

## Results

### Patient Demographics

There were 303 patients who attended the three MDTED clinics over the study period. After applying the exclusion criteria, 49 patients were excluded from the study as they did not have a diagnosis of TED; 15 patients had inadequate records and 3 were seen at other hospitals where we could not access their endocrine records. Consequently, 236 patients were included in this study; 106 were at CMH, 82 at the WEH, and 48 at CXH. The baseline patient demographic data are shown in **Table 2**. Out of the 13.6% (32/236) patients who were diabetic: 12.5% (4/32) were type 1, 84.4% (27/32) type 2 and one patient had diabetes secondary to thyroid hormone resistance syndrome with a G332E mutation.

**Table 2:** Patient demographics across 3 multidisciplinary clinics.

	All TED group		Central Middlesex Hospital		Western Eye Hospital		Charing Cross Hospital	
	*n = 236*		*n = 106*		*n = 82*		*n = 48*	
	***^n^***		***^n^***		***^n^***		***^n^***	
**Age at first TED clinic(yrs)**	**236**		**106**		**82**		**48**	
Median (IQR)		49 (36-57)		43 (33-55		52 (43.8-60)		48 (34.5-54.8)
Range		18 - 82		18 - 82		23 - 82		23 - 77
**No. (%) Female**	**236**	183 (77.5)	**106**	83 (78.3)	**82**	63 (76.8)	**48**	37 (77.1)
**Ethnicity**	**204^a^**		**99^b^**		**69^c^**		**36^d^**	
No. (%) Caucasian		71 (34.8)		25 (25.3)		26 (37.7)		20 (55.6)
No. (%) Afro-Caribbean		55 (27.0)		36 (36.4)		12 (17.4)		7 (19.4)
No. (%) Asian		46 (22.5)		33 (33.3)		9 (13.0)		4 (11.1)
**Smoking status**	**223^e^**		**106**		**69^c^**		**48**	
No. (%) Current smokers		52 (23.1)		26 (24.5)		13 (18.8)		13 (27.1)
No. (%) Ex-smokers		41 (17.9)		12 (11.3)		18 (26.1)		11 (20.8)
**No. (%) Positive family history**	**236**	74 (31.4)	**106**	34 (32.1)	**82**	23 (28.0)	**48**	17 (35.4)
**No. (%) Diabetes**	**236**	32 (13.6)	**106**	11 (10.4)	**82**	14 (17.1)	**48**	7 (14.6)
**Thyroid status at first clinic**	**231^f^**		**102^g^**		**81^h^**		**48**	
No. (%) Euthyroid		110 (47.6)		57 (55.9)		29 (35.8)		24 (50.0)
No. (%) Hyperthyroid		96 (41.6)		36 (35.3)		39 (48.1)		21 (43.8)
No. (%) Hypothyroid		25 (10.8)		9 (8.8)		11 (13.6)		5 (10.4)

^a^Data unrecorded n=32, ^b^data missing n=7, ^c^data missing n=13, ^d^data missing n=12, ^e^data missing n=13, ^f^data missing n=5, ^g^data missing n=4, ^h^data missing n=1.

Data recorded from inception of the clinic until January 2019; July 2012 to January 2019 for Central Middlesex Hospital, January 2016 to January 2019 for Western Eye Hospital and January 2017 to January 2019.

In bold: n refers to the number of patients (participants) which had the data available in their patient record notes for each demographic/endocrinology/ophthalmology characteristic.

### Timing of Referral

**Figure 1:**
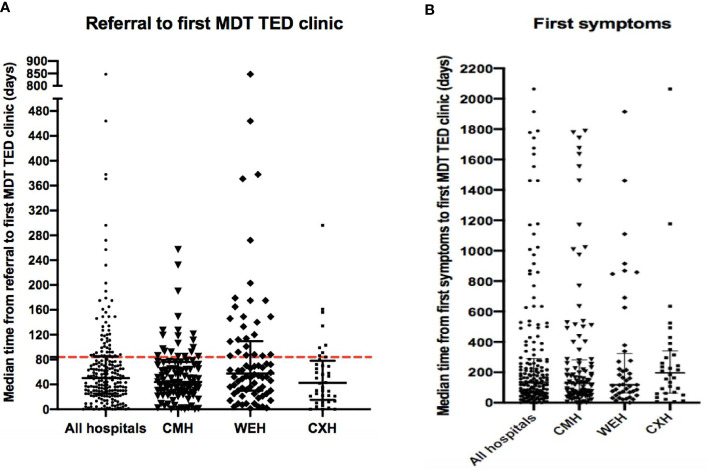
**(A)** Time from referral to first MDTED clinic. The median (IQR) time (days) from referral to date of first MDTED clinic. All hospitals n=215, CMH n=101, WEH n=74, CXH: n=40. Horizontal line represents the target time by TEAMeD-5 (84 days or 3 months). **(B)** Time from first symptoms to first MDTED clinic. The median (IQR) time (months) from patient reported onset of first symptoms of Graves’ Orbitopathy and the date of their first specialist multidisciplinary thyroid eye disease clinic. All hospitals n=177, CMH n= 101, WEH n= 44, CXH n= 32. Calculation from patient data available on symptom onset and referral time. CMH, Central Middlesex Hospital (part of London North West University Healthcare NHS Trust); WEH, Western Eye Hospital; CXH, Charing Cross Hospital.

After referral, 75.2% (177/236) patients were seen in a MDTED clinic within three months of referral. The median time from the patients reported first onset of symptoms and date of their first MDTED clinic was 135 days (**Figure 1**).

### Management of Thyroid Dysfunction

The baseline thyroid status of the patients is shown in **Tables 3** and **4**. Patients who were euthyroid at their first MDTED appointment were the result of well controlled thyroid disease or euthyroid GD. Out of 166 patients who had TSH antibody titre measurement, 75.9% (126/166) had a positive titre status. In our cohort, 23.7% (56/236) patients had a recurrence (relapse) of hyperthyroidism; 66.7% (37/56) had this before and 33.3% (19/56) after their first attendance at the MDTED clinics. One hundred and three patients were discharged from the MDTED clinics during the study period and 76.7% (79/103) were biochemically euthyroid at discharge.

**Table 3:** Patient baseline characteristics by disease activity at presentation.

	Cohort 1		Cohort 2		Cohort 3		Cohort 4	
	(CAS 0 - 1)		(CAS 2)		(CAS ≥ 3 and non-sight threatening)		(DON)	
	*n = 127*		*n = 31*		*n = 44*		*n = 17*	
	***^n^***		***^n^***		***^n^***		***^n^***	
**Age (yrs)**	**127**		**31**		**44**		**17**	
Median (IQR)		48 (35.0 - 57.0)		40(31.0 - 49.0)		49.0 (35.5 - 53.5)		57.0 (53.0 - 71.5)
Range		18 - 82		21 - 73		25 - 76		35 - 80
**No. (%) Female**	**127**	105 (82.7)	**31**	21 (67.7)	**44**	32 (72.7)	**17**	12 (70.6)
**Ethnicity**	**104^a^**		**28^b^**		**42^c^**		**15^c^**	
No. (%) Caucasian		36 (34.6)		7 (25.0)		16 (38.1)		6 (40.0)
No. (%) Afro-Caribbean		26 (25.0)		9 (32.1)		14 (33.3)		3 (20.0)
No. (%) Asian		25 (24.0)		10 (35.7)		7 (16.7)		2 (13.3)
**Smoking status**	**118^d^**		**30^e^**		**42^c^**		**17**	
No. (%) Current smokers		28 (23.7)		9 (30.0)		10 (23.8)		2 (11.8)
No. (%) Ex-smokers		23 (19.5)		3 (10.0)		9 (21.4)		2 (11.8)
**No. (%) Positive family history**	**127**	39 (30.7)	**31**	12 (38.7)	**44**	18 (40.9)	**17**	3 (17.6)

^a^Data missing n= 23, ^b^data missing n= 3, ^c^data missing n=2, ^d^data missing n=9, ^e^data missing n= 1.

Summary of 219 patients who had a recorded baseline clinical activity score (CAS) from July 2012 to January 2019.

DON, Dysthyroid Optic Neuropathy (sight-threatening disease).

In bold: n refers to the number of patients (participants) which had the data available in their patient record notes for each demographic/endocrinology/ophthalmology characteristic.

**Table 4:** Endocrinological characteristics of the cohort by disease activity.

	Cohort 1		Cohort 2		Cohort 3		Cohort 4	
	(CAS 0 - 1)		(CAS 2)		(CAS ≥ 3 and non-sight threatening)		(DON)	
	*n = 127*		*n = 31*		*n = 44*		*n = 17*	
	***^n^***		***^n^***		***^n^***		***^n^***	
**Positive autoantibody**								
No. (%) TSH	**90^a^**	59 (65.6)	**24^b^**	20 (83.3)	**30^c^**	28 (93.3)	**10^d^**	7 (70.0)
No. (%) TPO	**64^e^**	31 (48.4)	**20^f^**	8 (40.0)	**23^g^**	13 (56.5)	**7^h^**	3 (42.9)
**TSH antibody titre (AU/mL)**	**90^a^**							
Median (IQR)		4.2 (1.1 -13.7)	**24^b^**	12.3 (3.6 - 20.9)	**30^c^**	15.4 (5.0 - 29.7)	**8^i^**	4.0 (1.2 - 14.4)
Range		0.5 - 100.0		0.7 - 59.6		0.3 - 56.8		0.9 - 15.0
**TPO antibody titre (AU/mL)**								
Median (IQR)	**46^j^**	121.5 (12.7 - 357.2)	**20^f^**	152.0 (5.3 - 285.0)	**23^g^**	196.0 (20.0-491.2)	**7^h^**	56.5 (31.1 - 83.2)
Range		2.0 - 2379.0		1.0 - 617.0		1.0 - 879.0		25.8 - 88.9
**Free T3 (pmol/L)**								
Median (IQR)	**112^k^**	5.7 (3.8 - 12.7)	**30^l^**	7.8 (4.0- 15.5)	**43^l^**	7.3 (4.4 - 22.6)	**13^m^**	7.1 (4.8 - 14.9)
Range		0.4 - 46.2		1.6 - 46.1		0.9 - 47.1		0.9 - 46.1
**Thyroid status at first clinic**	**122°**							
No.(%) Hyperthyroid	36 (29.5)	**31**	18 (58.1)	**44**	26 (59.1)	**16^l^**	7 (43.8)
No. (%) Hypothyroid	15 (12.3)		2 (6.5)		6 (13.6)		1 (6.3)
No. (%) Euthyroid	71 (58.2)		11 (35.5)		12 (27.3)		8 (50.0)
**No. (%) Diabetes**	**127**	15 (11.8)	**31**	3 (9.7)	**44**	7 (15.9)	**17**	4 (23.5)
**HbA1c (mmol/l)**	**72^p^**							
Median (IQR)		38.2 (36.0 - 42.0)	**20^f^**	38.0 (35.3 - 40.8)	**31^q^**	40.0 (36.0 - 45.0)	**14^r^**	42.0 (36.0 - 45.3)
Range		26.0 - 48.0		33.0 - 66.0		30.0 - 77.0		35.0 - 61.0
**Thyroid medication**	**87^s^**							
No. (%) Carbimazole (block and replace)		12 (13.8)		5 (23.8)		6 (16.2)		1 (7.7)
No. (%) Carbimazole (titration)		58 (67.7)	**21^h^**	12 (57.1)	**37^d^**	23 (62.2)	**13^m^**	9 (69.2)
No. (%) PTU		9 (10.3)		1 (4.8)		5 (13.5)		1 (7.7)
No. (%) Thyroxine		8 (9.2)		3 (14.3)		3 (8.1)		2 (15.4)
**No. (%) Previous radioiodine**	**127**	11 (8.7)	**31**	2 (6.5)	**44**	5 (11.4)	**17**	1 (5.9)
**No. (%) Thyroidectomy**	**127**	19 (16.8)	**31**	1 (3.2)	**44**	7 (14.3)	**17**	0 (0.0)

^a^Data unrecorded n=37, ^b^data unrecorded n=7, ^c^data unrecorded n=14, ^d^data unrecorded n=7, ^e^data unrecorded n=63, ^f^data unrecorded n= 11, ^g^data unrecorded n= 21, ^h^data unrecorded n= 10, ^i^data unrecorded n= 9, ^j^data missing n= 81, ^k^data missing n=15, ^l^data missing n= 1, ^m^data missing n= 4, ^o^data missing n= 5, ^p^data unrecorded n= 55, ^q^data unrecorded n=13, ^r^data unrecorded n= 3, ^s^data missing n= 40.

Summary of the endocrinological characteristics stratified by disease activities as measured by clinical activity score (CAS).

DON; Dysthyroid Optic Neuropathy (sight-threatening disease), PTU; Propylthiouracil, TPO; Thyroid Peroxidase, TSH; Thyroid stimulating Hormone.

Normal ranges: TSH: <0.4AU/mL, TPO: <75 AU/mL, free T3: 2.5-5.7 pmol/L.

In bold: n refers to the number of patients (participants) which had the data available in their patient record notes for each demographic/endocrinology/ophthalmology characteristic.

### Radioiodine Treatment (RAI)

Twenty-nine patients received RAI treatment; 72.4% (21/29) had the treatment before the diagnosis of TED. Two of the eight patients with an established TED diagnosis had adjunctive oral steroid cover during their RAI treatment. The others were deemed to be low risk for eye disease progression by the MDT team, therefore were not prescribed oral steroid cover.

### Smoking and Selenium

Baseline smoking status, where it was documented for patients is shown in **Table 2**. All current smokers received documented smoking cessation advice. Out of the 135 new patients seen after the introduction of TEAMeD-5 in 2017, 60.0% (81/135) received documented advice regarding the potential benefits of selenium supplementation.

### Activity and Severity of TED

Two hundred and nineteen patients (91.5%) had a documented CAS at their first MDTED clinic attendance; 20.1% (44/219) were had a CAS of 3 or more. In our cohort, 7.2% (17/236) had sight-threatening disease (dysthyroid optic neuropathy and/or sight-threatening corneal exposure) (**Tables 3** and **4**).

### Investigations and Treatment

Seventy percent (165/236) had one or more orbital Magnetic Resonance imaging (MRI) scans; 57.6% (95/165) were reported to have radiological evidence of orbital inflammation on their baseline scan (either qualitatively with STIR sequences or quantitatively with non-echo planar diffusion weighted imaging - DWI).

As first-line immunosuppression treatment, 33.9% (80/236) received intravenous methylprednisolone (IVMP), as recommended by the EUGOGO consensus (**Table 1**). The median time (range) from decision to treat and treatment initiation was 7 (1-90) days. In our cohort, 12.7% (30/236) received second-line immunosuppressant therapy usually with mycophenolate mofetil (Cellcept). Further, 16.1% (38/236) patients required orbital radiotherapy (OR). Twenty-five of these patients’ notes showed time of referral and date of treatment; the median time (range) from referral to OR was 30.5 (7-70) days. Twenty-five (10.6%) patients underwent orbital decompression surgery during the follow-up period, of these nine (36.0%) were emergency decompressions as adjunctive treatment for sight-threatening disease (median decision to treatment time: 1.6 days, range 0-6). All our 17 patients with sight-threatening disease started their sight preserving treatment within 2 weeks of diagnosis (**Table 1**).

## Discussion

The concept of combined multidisciplinary clinics has been well established in many disciplines to harness best available expertise to optimise patient management and outcomes. It has also been shown to improve the time to diagnosis and treatment in TED (5, 6, 8). Endocrinologists and ophthalmologists simultaneously seeing patients in these MDT clinics, ensures that a consistent and coherent management plan is clearly communicated and discussed with the patient and any concerns promptly addressed with readily available subspecialist expertise. The inception of two of our clinics at CMH (2011) and WEH (2015) predate the publication of TEAMeD-5 (2017) and the BOPSS audit criteria (December 2019). Data collection for this study predated the publication of the BOPSS criteria. Therefore, our multi-centre retrospective study of three MDTED clinics can be interpreted as a ‘real world’ snapshot of the MDT management of thyroid eye disease prior to introduction of established TED standards in a metropolitan multi-ethnic setting.

We had a similar age range and female:male ratio to other published multi-centre audits (9, 10). Our ethnically diverse cohort was comprised of 49.5% Afro-Caribbean or South Asian patients and this reflects the multi-ethnic London population. Previous audits of MDTED clinics have been less comprehensive in describing the ophthalmological and endocrinological profile of their patients (9, 10). Our prevalence of sight-threatening disease of 7.2% is higher compared to many other studies (11–13). We also had a higher number of patients requiring immunosuppression; 33.9% and 16.2% of our cohort received IVMP and orbital radiotherapy respectively compared to the PREGO study (23.0% and 6.0%), possibly reflecting a cohort with more severe disease (13). Only 75.9% of our cohort had a TSH-receptor positive titre, demonstrating that TED is primarily a clinical (and radiological) diagnosis, although serological testing is useful to identify Graves’ Disease patients at risk (as per TEAMeD-5) (6). Most of our patients who received RAI did so prior to attending a MDTED clinic. There may be a very long gap between date of RAI treatment and onset of TED, so it is difficult to deduce whether RAI has contributed to the TED (14). However, out of those who received RAI after an established diagnosis of TED, only a quarter had prophylactic oral steroid cover. Oral steroids have a range of undesirable side effects for example weight gain, diabetes, and osteoporosis (15). These adverse effects must be considered before prescribing steroids, especially in patients deemed to be low risk for eye progression. There may also be a trend that with increasing awareness of TED, pre-RAI TED screening may account for increasing referrals to eye services. It is also encouraging that some of the key initiatives of TEAMeD-5 have already been implemented with 100% smoking cessation advice given to current smokers in our cohort. There is also increasing numbers (almost two-thirds of our cohort) who have received documented advice about selenium supplementation. In our experience, many patients have started selenium supplementation by the time of their first attendance at the MDTED clinic usually on the advice of their endocrinologists.

### Limitations

This is a retrospective descriptive study at a single time point prior to the introduction of established TED standards. It would be interesting and informative to repeat this service evaluation in the future to evaluate for changes in practice after the TED standards have become embedded.

Data on every variable could not be recorded due to missing information from patients’ notes. TSH-R measurement was less widespread prior to 2015, therefore only 70.3% of our patients had a recorded TSH antibody titre. In our cohort, there were some patients who had clinically active TED but a low CAS. CAS, having been developed in a predominantly white Dutch TED cohort, in our experience underestimates activity in patients with a high Fitzpatrick skin type due to its reliance on soft tissue appearance, such as erythema, and this is pertinent in a cohort of high ethnic diversity. The CAS scoring system places a greater weight on symptoms resulting from acute orbital congestion, as these comprise 5 of the total 7 points which can be allocated on the first visit. Ocular muscle involvement is not reflected in the scoring system, neither is posterior orbital pathology (16). Highly relevant signs such as diplopia at the first visit are also not included in the CAS scoring system (17). Therefore, changes in visual function are not scored for in the first visit where CAS is scored out of 7, so patients can have severe ocular motility restriction and decreased acuity that is not scored for in this setting (17). Recently, non-echo planar diffusion weighted imaging (DWI) MRI has been evaluated as a valuable tool, in adjunction with CAS, for investigating posterior orbital disease in TED and in providing higher resolution orbital images (16, 17). This imaging has been an invaluable adjunct for TED patient management in our clinics for the past eight years. Nevertheless, CAS also has a strong inter-specialty understanding and is used interchangeably by endocrinologists and ophthalmologists. Our aim with our ongoing collaboration alongside our radiological colleagues is to develop a Radiological Activity Score (RAS) to complement CAS to enable a more comprehensive assessment of disease activity and severity. It is well recognised that TED can significantly impact on Quality of Life (QoL) (18–21). QoL measurement with a validated instrument such as the GO-QOL, which is the gold standard in assessing the impact of TED, is recommended as part of the ongoing clinical assessment (19–21). However during the study period, the regular use of the validated GO-QOL questionnaire remained inconsistent in the aforementioned clinics.

### Thyroid Eye Care in the Post Covid-19 Landscape

This service evaluation was carried out before the Covid-19 pandemic. In the height of the COVID pandemic, all routine face to face MDT clinic appointments were suspended. We stopped all our patients undergoing intravenous steroid treatment for non-sight-threatening disease and a significant proportion relapsed meaning we had to resume immunosuppression generally in the form of oral steroids during the lockdown period. In practice TED management is complex and relies on many face to face interactions such as orthoptic measurements, blood tests and ophthalmic and systemic assessments and we have found that a significant proportion of patients had significantly deteriorated despite reporting stability over a virtual consultation. The thyroid MDT clinic was one of the first, with the support of our management, to resume face to face activity for all patients once the height of the pandemic subsided and decisions regarding starting or resuming immunosuppression were made on the basis of these face to face consultations. With the investment of National Health Service (NHS) information technology in the UK, there may be a role for virtual MDT-lite clinics, which can enable more patients to have access to this modality, especially in parts of the country where there is significant geographical separation between endocrine and ophthalmic services. MDT clinics reduce duplication of appointments, avoids patients attending hospitals twice for the same condition and offers a consistent management strategy with a wholistic approach. We recognise however in a post COVID healthcare landscape, there will be significant fiscal constraints as regards service developments.

### Implications for Future Work

There is consensus that TED management should be safe, timely, efficacious, equitable and patient-centred. In the UK, TEAMeD has worked closely with BOPSS to set up sustainable targets (7). Regular auditing of key performance indicators (KPIs) is of paramount importance. One of our recommendations is the set up of a dedicated electronic patient record platform to record this data prospectively in the clinic setting to avoid an onerous audit process, with a system to enable continuous and effortless GO-QOL evaluations at every consultation. The MDT service would also benefit from having dedicated co-ordinators as presently this administrative burden is mainly borne by clinical staff. This would be highly beneficial to provide more patient-centred care. We highlight the important of signposting patients to support organisations for additional support. In the UK, these include the British Thyroid Foundation and Thyroid Eye Disease Charitable Trust (TEDct). Ideally patients should have access to psychological counselling in view of the burden of significant disfigurement from TED. We believe that our approach, integrating TEAMeD-5 audit standards with the BOPSS recommendations forms a more complete service evaluation model for future MDTED services to implement the aims of the Amsterdam Declaration on Graves Orbitopathy. The clinical findings of our service evaluation in a multi-ethnic metropolitan population may not be widely applicable to all TED populations. Our main aim is to show the feasibility of producing key performance indicators for benchmarking TED care in a multi-disciplinary setting. We would look forward to other centres with a different cohort demographic to conduct a similar service evaluation to establish the range of TED presentations in an MDT setting designed to enable earlier diagnosis and treatment of TED.

## Data Availability Statement

The raw data supporting the conclusions of this article will be made available by the authors, without undue reservation.

## Ethics Statement

The studies involving human participants were reviewed and approved by Imperial College Audit Office. Written informed consent for participation was not required for this study in accordance with the national legislation and the institutional requirements.

## Author Contributions

VL conceived and designed the study. SF acquired, analysed, and interpreted the data. SF, CF, and VL drafted the manuscript. RA, RJ, AA, VB, and KM contributed to the manuscript. All authors contributed to the article and approved the submitted version.

## Funding

Study funding was provided by Imperial College London as part of SF Bachelor of Science work.

## Conflict of Interest

VL is the British Oculoplastic Surgery Society National Lead for TEAMeD (UK Thyroid Eye Disease Amsterdam Declaration Implementation Group).

The remaining authors declare that the research was conducted in the absence of any commercial or financial relationships that could be construed as a potential conflict of interest.
